# Parents' perception of stroller use in young children: a qualitative study

**DOI:** 10.1186/s12889-015-1989-6

**Published:** 2015-08-20

**Authors:** Catherine S. Birken, Bradley Lichtblau, Talia Lenton-Brym, Patricia Tucker, Jonathon L Maguire, Patricia C. Parkin, Sanjay Mahant, on behalf of the TARGet Kids! Collaboration

**Affiliations:** Hospital for Sick Children, Toronto, ON Canada; Child Health Evaluative Sciences, The Hospital for Sick Children Research Institute, Toronto, ON Canada; Applied Health Research Centre, Li Ka Shing Knowledge Institute, St. Michaels Hospital, Toronto, ON Canada; Departments of Pediatrics, Hospital for Sick Children, Toronto, ON Canada; Faculty of Medicine, University of Toronto, Toronto, ON Canada; Michael G. Degroote School of Medicine, McMaster University, Hamilton, ON Canada; Department of Pediatrics, St. Michael’s Hospital, Toronto, ON Canada; School of Occupational Therapy, University of Western Ontario, London, ON Canada

**Keywords:** Child, Preschool, Infant equipment, Stroller, Motor activity, Physical activity

## Abstract

**Background:**

Despite their wide usage, it has recently been suggested that stroller use may reduce physical activity levels of young children. However, there have been no studies on stroller use as it relates to physical activity outcomes. The objectives of this study were to understand the context of stroller use for young children and parents’ perceptions of the relationship between stroller use and their children’s physical activity.

**Methods:**

Parents of children 1 to 5 years of age were recruited through two sites of TARGet Kids!, a primary-care, practice-based research network in Toronto, Canada. Fourteen semi-structured interviews were conducted. Interviews were audio recorded and transcribed verbatim and two independent reviewers conducted thematic analysis. A number of strategies were employed to ensure the trustworthiness of the data.

**Results:**

Parents discussed reasons for stroller use (i.e., transportation; storage; leisure; supervision/confinement; parent physical activity; and sleep), factors that influence the decision to use a stroller (i.e., caregiver choice; convenience, timing, distance; family lifestyle; and child preference), and perceived impact of stroller use on physical activity (i.e., most parents did not recognize a connection between stroller use and physical activity).

**Conclusion:**

This study provides a context for researchers and policy makers to consider when developing stroller related physical activity guidelines for young children.

## Background

Strollers are widely used for the easy transport of children. According to *Consumer Reports*, approximately 5.6 million strollers were sold in the United States in 1998 [1]. Strollers have recently been conceptualized, however, as sedentary devices that restrict movement and reduce opportunities for physical activity. Obesity is a growing concern for young children. In Canada, 21.5 % of children aged 2–5 years are overweight [2] and studies in the United States suggest that obesity before age 5 strongly predicts future obesity [3]. Physical activity is important for healthy growth, social and motor development in children [4]. To encourage habits that decrease the risk of obesity in young children, The American Academy of Pediatrics (AAP) and the Canadian Society for Exercise Physiology (CSEP) have published guidelines which include recommendations to limit stroller use for young children [5–7]. The AAP guidelines recommend reducing sedentary transportation by car or stroller for 4-6 year-old children, and the CSEP guidelines recommend limiting prolonged sitting or being restrained (e.g., stroller, high chair) for more than 1 h at a time in 0-4 year-old children. Researchers have suggested that restraining children in strollers and other confined seats is a risk factor for limited physical activity [8].

Limited research exists on prevalence, predictors, and health outcomes related to stroller use, with the exception of stroller-related injuries [9]. In fact, no studies were found on this topic in our own literature review examining stroller use and physical activity and obesity outcomes. It is unknown, therefore, if stroller use is associated with reduced or increased physical activity. For example, we hypothesize that in some cases, stroller use may increase accessibility to areas intended for physical activity, such as parks. This hypothesis requires further research – specifically, a qualitative exploration of the contexts of stroller use in families with young children, and parents’ perceptions of health outcomes related to stroller use. In light of the paucity of research available, the primary purpose of this study was to understand the context in which strollers are being used with young children. A secondary objective was to examine how parents perceive the relationship between stroller use and their children’s physical activity.

## Methods

Fourteen semi-structured interviews were conducted with parents of young children recruited through two sites of TARGet Kids!, a primary-care, practice-based research network in Toronto. English-speaking parents of ambulatory children aged 1–5 years already recruited to TARGet Kids! were approached at their child’s well-child visit, as per the TARGet Kids! research protocol, [10] and written informed consent for participation in the study was obtained from parents. Recruitment procedures and inclusion criteria for TARGet Kids! are detailed elsewhere [9]. We used a convenience sampling technique, and recruited participants from two TARGet Kids! primary care practice sites between January 2012 to August 2012. These two sites were chosen as they were located in two distinct urban neighbourhoods in Toronto, and had adequate space to conduct qualitative interviews. Participants of TARGet Kids! were not participating in any TARGet Kids! related intervention studies that pertain to physical activity prior to or during the time of recruitment for this study.

Interviews lasted approximately 20 min, were completed immediately preceding or following the child’s well-child visit, and were audio recorded and transcribed verbatim. The semi-structured interview questions included stroller use (how and when parents use a stroller), barriers and facilitators of stroller use, and the relationships between stroller use and physical activity and health. TARGet Kids! researchers developed and pilot-tested the interview questions. One researcher conducted, recorded, and transcribed each interview (BL). Two independent reviewers completed a thematic analysis using the method described by Braun and Clarke (BL, CSB) [11]. Each reviewer developed an independent set of codes based on the interview transcripts, which were then categorized into overarching themes. Both reviewers met multiple times throughout the study, and met with a third reviewer (SM) to review the ongoing analysis and to generate an overall map based on the discussed themes. A number of strategies including member-checking, multiple debrief meetings following interviews, detailed documentation of the research process for transferability, as described by Guba and Lincoln, were employed throughout the data collection and analysis phase to ensure unbiased findings and trustworthiness of the data [12]. The study was completed with the approval of the Research Ethics Board at The Hospital for Sick Children in Toronto, Canada.

## Results

Participants included parents who had least 1 child aged 1-5 years of age participating in TARGet Kids!. The mean (SD) age of the children that were the focus of the interview were 31 (17) months; they had a total of 12 siblings with a mean (SD) age of 67 (49) months. 3 of the 14 participants were fathers. Thirteen of the children (93 %) had mothers with college/university-level education and the remaining mother had an apprenticeship/trades certificate/diploma. During the interviews, participants discussed their stroller use and how stroller use impacted their lifestyle. The themes that emerged from these discussions fall under three main categories: (1) purposes of stroller use; (2) factors that influence the decision to use a stroller; and, (3) perceived impact of stroller use on physical activity. After 14 interviews, we felt we had reached theoretical saturation in the themes that emerged. Please see Tables 1-3 for example quotes for each of these themes.

**Table 1:** Parent comments regarding purposes for stroller use

Transportation	“The nanny wheels him around to play dates and to social activities and to the park and I put him in the stroller when I’m in a rush going somewhere walking to get groceries or something.”
	Its {stroller use} faster and because of timing of eating sleeping; its about time limits and getting things done.
Storage	“I bring the stroller pretty much everywhere I go… it usually comes along just to carry all the stuff that we need to carry.”
	I just bring the stroller so that I can put my stuff in it pretty much”
Leisure	“to have time outdoors.”
	“…On the weekend we go for family walks to the park.”
	“…if we go somewhere like the zoo.”
Supervision/ confinement	“Safety definitely is a concern and traffic is a concern its very busy.”
Parents’ physical activity	“When I go for exercise walks… I push him because then my focus is on me getting exercise and not him.”
Sleep	“….or like when we went away on holiday sp we couldn’t really give him a nap so we really encouraged him to be in the stroller for about an hour to rest…

### Purposes of stroller use

Parents reported six different reasons for using strollers. Each interviewee listed at least one of the six rationales, although many stated that they use strollers for multiple purposes:

- ***Transportation.*** Parents reported that they use strollers as a means of transportation, both to facilitate walks and as a means to transport their child(ren) to a specific destination or activity. In some cases, the stroller was used as an alternative to taking public transportation or an automobile. When asked about how they use the stroller, one parent said, “usually just to get anywhere, popping out [for] groceries… heading to the park… basically anytime we need to get somewhere with a fairly tight timeline”.
- ***Storage.*** Additionally, strollers provide room for the storage of items that parents often travel with when on the move with young children, including diaper bags, food, extra clothing, shopping items, and other supplies that they may need throughout the day. Many parents noted that the storage capacity of strollers is one of its main conveniences. As one parent reinforced, “…you can’t go to the park without taking a blanket and water and snacks for the kids… I would have… a travel backpack[like] a navy leader if I was going without a stroller”. Even when not entirely necessary for transporting the child, this parent explained that they might choose to take a stroller with them on walks because of its carrying capacity.
- ***Leisure.*** Strollers provide an opportunity for both child and caregiver to spend time outside for leisurely walks. Parents described this use as beneficial in that it allows the child to get fresh air and explore the neighbourhood. One parent said that the stroller gives the child an opportunity for “…quiet time in the day to just sort of enjoy the fresh air… [and] to have time outdoors”.
- ***Supervision/Confinement.*** Strollers allow caregivers to safely supervise their children. Interviewees noted that there are certain circumstances, when running errands, for instance, in which they want the child to be restrained in order to enable direct supervision. As one parent explained, “[y]ou can do a lot of things when the child is in the stroller. You can [do] a little bit of grocery shopping, go get a haircut, and [you’re] in control”.
- ***Parent Physical Activity.*** Caregivers described that they sometimes take their children in strollers for long walks or push the stroller while jogging or running as a means of improving their own health and well being. Interviewees noted that this allows them to achieve two objectives: their children are able to be outdoors and get fresh air while the parent can engage in their own physical activity objectives. In some instances, a stroller was purchased for its function as a running device. One parent explained, “…[stroller use] was my means of getting exercise where I would run and he would have some sort of downtime… I would let him fall asleep in the jogger if I was running”.
- ***Sleep.*** Strollers provide a setting for sleep when away from home. One parent gave an example: “[w]hen we go on family walks… if it’s going to cross over her nap time, she’ll get in the stroller and have a nap…”. The interviewees noted that the use of a stroller as a place to sleep is particularly conducive for longer walks or daytrips, so that their children are able to maintain their normal sleep patterns throughout the day.

### Factors that influence the decision to use a stroller

Parents discussed both parent and child influences on the day-to-day decision to use a stroller. There are three main categories that summarize the decision-making factors surrounding stroller-use: (1) caregiver choice, based on convenience, timing, and distance; (2) family lifestyle; and, (3) child preference (see Table 2).

**Table 2:** Factors that influence the decision to use a stroller

Caregiver preference	“I think kids would probably prefer to run around or ride a bike if they were given the option. I think it’s more parents who enforce the strollers if they want to get somewhere faster…I think it’s more the parents aren’t patient…because I know I’m certainly that way sometimes.”
Time constraints	“It takes too much time in the morning to walk the kids for 15 min with them walking themselves, we’d never make it.”
	“Sometimes we will put her in it [a stroller] more often, because it’s easier to kind of cart her somewhere strapped in than waiting for her to walk.”
Family lifestyle	“Stroller use definitely does depend if the parent is working or not and how many hours a day they are working…if the child is going to daycare or not, if the parent is primarily taking care of the child. Things could be different if it’s a babysitter, it’s different if you’re working morning or evenings…it just all depends on how one person’s life is. I would think that would affect the stroller use completely.”
Child walking ability	“… if it were short distances like walking into her daycare from the care I mean I would carry her or she would walk. I would not bother with the stroller for probably distances of up to 200 metres, 150 metres.”
	“I think when they could walk reasonably well without you know doing all the banging and tripping and on their knees [that would be time to stop using a stroller].”
Child preference	“…I think she genuinely likes to explore and walk and have fun.”
	“…I think it’s child-dependent though because my oldest two, they were more content in the stroller to just look around, and then (name of child) was more interested in wondering around on his own.”
	“… she doesn’t really like to sit in it that much, so like if we’re in the neighbourhood and we’re just going shopping… or we’re just going to the park she’s usually just walking or at least 75 % of that…”
	“I think when he got to that point where he was… walking and interested in the walking and exploring I don’t think I ever fought that…”

Parents noted three key benefits of using a stroller: convenience, timeliness, and ability to travel a further distance than would otherwise be feasible. Strollers were considered a more convenient alternative to parents carrying their child(ren) when they no longer want to walk. Additionally, when caregivers had limited time, they might use the stroller instead of having the child(ren) walking because it is faster. One parent explained, “[i]f we wanted to get somewhere in a decent [amount of] time we put her in the stroller, because we have done the walk, you know, a couple of blocks and that takes some time, it’s slow”. Distance to the desired destination was also an important factor in deciding whether to use a stroller. For a caregiver to decide to have the child walk, the distance had to be appropriate for the child’s ability and willingness at the time. Referring to taking her daughter to swimming lessons, one parent explained, “…it was up a steep hill and it’s still a good 20 min walk even for me and she could usually make it to swimming but by the time swimming’s done and she’s cold and cranky and it’s about 5:30…we regularly used a stroller…just to get her home quickly”. The children’s developmental stage in walking ability influenced these factors, as the caregivers would let their children walk as far as feasible until the child grew tired. When asked how she decided to stop using the stroller, one parent explained, “I think when they could walk reasonably well without…doing all the banging and tripping and on their knees then… [walking is] good practice for them and keeps them healthy…”. Another described bringing a stroller along on a walk even though the child would walk for the majority of the trip, “…when you’re out, eventually they do start to get a little tired so I kind of use it as a method to get her home quickly so we can get to the nap”.

In addition, caregivers sometimes based their decision on their individual lifestyle philosophies. This was sometimes a preference to limit the use of automobiles, in which case the use of a stroller replaced the need to drive or take public transit to get to a further destination and in other cases a desire to spend extended periods outdoors with the family.

Some parents noted that they would often let their children decide whether or not they wanted to use a stroller. In these instances, the stroller would be brought on a walk in case the child expressed a preference to use the stroller. One parent explained…”…if I think he is on the tired side {after school} and he’d rather just stroller home rather than walking, the walk is about 4 blocks, and sometimes he is not, and I bring it {the stroller} and we use if for the backpacks and he just walks”. Some reasons the parents reported that their child may express a preference included the child’s level of fatigue, hunger, mood, or desire to explore. Children sometimes chose to push the stroller themselves.

In addition to the main reasons caregivers identified using a stroller, convenience, timing and distance, lifestyle factors, and child preferences also influenced the decision. Some parents described how parents’ preference might play a role: “I think it’s more parents who enforce the strollers if they want to get somewhere faster… I think it’s more the parents [who] aren’t patient… because I know I’m certainly that way sometimes”. Other parents described the convenience of not having to carry their child, the inability of their child to walk long distances without tiring, and a lifestyle preference to spend more time walking over other forms of transportation such as car use, even if that meant having to use a stroller. Parents also described their children’s preferences to use a stroller, and suggested that those preferences may be related to the child’s own internal temperament, as suggested by the following quotations from parents: “…I think she genuinely likes to explore and walk and have fun,” and “…I think it’s child-dependent though because my oldest two, they were more content in the stroller to just look around, and then (name of child) was more interested in wondering around on his own”.

### Perceived impact of stroller use on physical activity

There was no consensus among parents on the relationship between stroller use and physical activity (see Table 3). After being presented with the sedentary behaviour guidelines recommending limited stroller use, many caregivers commented that they believed that the use of strollers reduced physical activity and commended the principles. However, caregivers also felt that strollers can, in fact, lead to increased levels of physical activity among children and families in certain circumstances. Most did not perceive any connection between stroller use and physical activity.

**Table 3:** Parent comments regarding the perceived relationship between physical activity and stroller use

Leads to increased physical activity	“…if there was not stroller available… you would be less likely to walk somewhere with your child, less likely to take them outside, [and] more likely to drive them.”
Leads to decreased physical activity	“I think kids would probably prefer to run around or ride a bike if they were given the option.”
	“I feel if they’re out of the stroller they… are more free… if they weren’t in the stroller… they would be… more physically active even if they were crawling.”
No connection with physical activity	“…I don’t really think there’s anything wrong with walking them in the stroller to the park and letting them run around there and then walking them home in the stroller.”
	“…I wouldn’t have really thought about it [connection with physical activity], its just part of what we do as a family.

Some caregivers explained that when children sit in a stroller, they are missing an opportunity to be physically active. One parent explained, “[c]ertainly there are mornings, like this morning when I took him to daycare, I said to myself, ‘wouldn’t it be nice if I could just strap him in the stroller and get us there in like good time?’ But then he doesn’t get to… explore and walk and stuff like that as we go to daycare so I guess it’s not as fun for him being in the stroller, so we try not to use it for that reason”. As is evident in this quotation, most caregivers who discussed strollers as barriers to physical activity did so in theoretical terms, and did not express that it was characteristic of their own experience.

Some parents felt that using a stroller more often may promote physical activity of the parents. One parent said that “…if there was not stroller available… you would be less likely to walk somewhere with your child, less likely to take them outside, [and] more likely to drive them”. Strollers were sometimes used specifically to promote physical activity by using it to transport the child to a setting more conducive to physical activity, such as a park. Parents felt that in such circumstances (for example the quote above regarding walking to swimming lessons and back), the child may have been able to walk instead of being transported in a stroller, but once they arrived, would be more tired and less physically active at the destination.

However, most interviewees believed that stroller use did not affect their children’s physical activity levels and reported that they ensure that their children are active. Caregivers reported that they chose to use strollers in instances where they would otherwise be carrying their children. One parent explained, “[i]f she weren’t in the stroller she’d get tired a bit more quickly and we’d end up carrying her, so I don’t really know that it makes much of a difference”. While parents recognized that, in theory, excessive stroller use may be detrimental to levels of physical activity, most explained that they did not perceive any correlation in their families. One parent said, “…there’s always the risk of having a child in their stroller for too long or for using it for the wrong reasons but if you’re not using it for the wrong reasons I don’t really see a problem with it”.

## Discussion

This is the first study to examine parents’ perceptions of stroller use in their families. Understanding parents’ perceptions of strollers in the context of physical activity is important in developing interventions and guidelines for parents around appropriate stroller use and physical activity for young children. The basis for children’s future physical activity behaviours is established early in life and physical activity in young children is influenced by parental behaviour [13]. Stroller use is a complex behaviour because it can be conceptualized as promoting both physical activity *and* sedentary behaviour in children. CSEP recently established Canada’s first physical activity and sedentary behaviour guidelines for children aged 0–4 years. They recommend limiting prolonged sitting or restraint, listing strollers as an example [6]. This recommendation is consistent with the Institute of Medicine’s physical activity guidelines for children aged 0–5 years, which advises caregivers to use strollers for toddlers and preschoolers only when necessary and to limit stroller use while infants are awake [14]. In a 2006 Policy Statement discussing the risk factors associated with childhood obesity, the AAP also highlighted excessive stroller use as a concern and recommended that stroller use be reduced for preschool-aged children [5]. The rationale for these guidelines is that children should participate in physically active forms of transport such as walking, as opposed to being sedentary in a stroller.

In the present study, parents report that strollers are used for a variety of reasons including transportation, storage, to promote leisure activities, for supervision and confinement, to facilitate parents’ physical activity, and as a setting for sleep. Guidelines to promote appropriate stroller use may have to consider the wide array of reasons for stroller use. Parents in this study believed that strollers had no detrimental effect on their child’s health, physical activity levels, or overall well being. Parents did not view strollers as primarily sedentary restraining devices that limited physical activity. Rather, parents described strollers as convenient methods of transportation that provided the opportunity for outdoor time as well as rest opportunities between activities, thus allowing their children to be more physically active. Physical activity in preschool children usually occurs during free play rather than during structured activities. Such play consists of short, intermittent bouts of activity with frequent rest periods [15]. Outdoor play is a key physical activity outcome measure in early childhood, and is associated with directly measured physical activity in this age group [16]. Parents perceived that their use of a stroller encouraged more time outdoors. Outdoor play has been shown to be associated with physical activity and other important child health benefits including motor development, vitamin D levels, and mental health [17]. It is important to note that when parents reflected on the relationship between strollers and physical activity, most explained that they did not perceive any connection in their own families. This is consistent with a previous qualitative study of parents of preschool aged children that found parents believed children are naturally physically active and there is little need for parental engagement to promote activity [18]. It may not be useful, therefore, for stroller related recommendations to be focused on physical activity related outcomes for children.

Parents reported that using a stroller facilitated their own physical activity as well. In Australia, stroller-walking groups have been proposed as a method to promote maternal well being and physical activity for mothers in the postpartum period. In a study reporting results of a survey on the perceived benefits and barriers associated with such stroller walking-groups, the purposes for parents using a stroller included walking to shops (84 %), walking for exercise (69 %), walking to visit friends (45 %), to calm the child (28 %), and other activities (24 %) [19]. Over 90 % of respondents said that walking with a stroller increases parent physical activity and mental well being. Clearly, the use of strollers may provide opportunities for parents’ health benefits (via walking, running, and stroller groups); however, these gains might be made at the expense of increasing sedentary behaviours of their young children.

It is important to note that parents reported using strollers as settings for sleep in their young children. While this might be a convenient choice for parents, current guidelines advise against using a stroller for sleep. For example, the Canadian Paediatric Society stated that “[c]ar seats and infant seat carriers must not replace the crib as a sleep surface due to the risk of the harness straps causing upper airway obstruction”. [20].

This is the first investigation to explore the use of strollers among young children, and reported on detailed interviews with 14 parents of young children. We are not aware of any published studies examining parents’ perception of stroller use, or the relationship between stroller use and physical activity outcomes in children. The principle limitation of the study is the generalizability of the findings. All parents in this study were recruited through two urban primary care sites as part of TARGet Kids!, a practice-based research network in Toronto, Canada. The education level of the sample of parents included in the study was high. Parental perception of physical environmental factors associated with stroller use may be different in non-urban settings, or in urban settings with different environmental characteristics such as sidewalks, traffic, safety, and public transportation. Most of the parents who participated in this study characterized themselves as mothers, and father’s perceptions of stroller use and physical activity may be different. Our sample size prevented us from commenting on how perceptions of stroller use may vary by child’s age. This is important as there may be different uses of strollers depending on the age and developmental stage of the child. In addition, current physical activity guidelines vary by child age [5, 6]. Despite these limitations, this study offers the first exploration of parent perceptions of the context, predictors, and physical activity outcomes related to stroller use in young children. It highlights the need for further research that expands the sample of interviewees to include parents of children of different ages, parents from diverse backgrounds and neighbourhoods, directly observes stroller use in young children, and use quantitative methods to assess stroller use and impact on physical activity and other related health behaviours in early childhood such as sleep.

## Conclusion

This study provides insights into parent perceptions of the context of stroller use, and demonstrates that parents may not consider the sedentary nature of strollers. Researchers interested in physical activity promotion in the early years might consider strollers and the context of their use in developing and testing strategies to promote physical activity and reduce sedentary behaviours. Strollers are commonly used device for young children and a better understanding of health impacts is needed. Finally, this study provides a contextual starting point for researchers and policy makers to consider when developing appropriate physical activity and sedentary behaviour guidelines for young children.

## Abbreviations

BMI: Body Mass Index
